# Identification of Single-Copy Orthologous Genes between *Physalis* and *Solanum lycopersicum* and Analysis of Genetic Diversity in *Physalis* Using Molecular Markers

**DOI:** 10.1371/journal.pone.0050164

**Published:** 2012-11-16

**Authors:** Jingli Wei, Xiaorong Hu, Jingjing Yang, Wencai Yang

**Affiliations:** 1 Beijing Key Laboratory of Growth and Developmental Regulation for Protected Vegetable Crops, Department of Vegetable Science, China Agricultural University, No. 2 Yuanmingyuan Xilu, Beijing, China; 2 The National Key Facilities for Crop Genetic Resources and Improvement, Institute of Crop Science, Chinese Academy of Agricultural Sciences, Beijing, China; United States Department of Agriculture, United States of America

## Abstract

The genus *Physalis* includes a number of commercially important edible and ornamental species. Its high nutritional value and potential medicinal properties leads to the increased commercial interest in the products of this genus worldwide. However, lack of molecular markers prevents the detailed study of genetics and phylogeny in *Physalis*, which limits the progress of breeding. In the present study, we compared the DNA sequences between *Physalis* and tomato, and attempted to analyze genetic diversity in *Physalis* using tomato markers. Blasting 23180 DNA sequences derived from *Physalis* against the International Tomato Annotation Group (ITAG) Release2.3 Predicted CDS (SL2.40) discovered 3356 single-copy orthologous genes between them. A total of 38 accessions from at least six species of *Physalis* were subjected to genetic diversity analysis using 97 tomato markers and 25 SSR markers derived from *P. peruviana*. Majority (73.2%) of tomato markers could amplify DNA fragments from at least one accession of *Physalis*. Diversity in *Physalis* at molecular level was also detected. The average Nei’s genetic distance between accessions was 0.3806 with a range of 0.2865 to 0.7091. These results indicated *Physalis* and tomato had similarity at both molecular marker and DNA sequence levels. Therefore, the molecular markers developed in tomato can be used in genetic study in *Physalis*.

## Introduction

*Physalis*, a member of the plant family Solanaceae, includes more than 90 species [1]. A number of species in the genus are of horticultural and economic importance due to their high nutritional value in vitamins content, minerals and antioxidants as well as potential medicinal properties including anti-bacteria, anti-inflammatory, and anti-cancer [2]–[9]. Some of the species such as *P. alkekengi* (Chinese lantern) can also be used for decoration. Therefore, the commercial interest in this genus has grown in many regions of the world in recent decade.

High variation of morphological characteristics have been observed and used to identify species in *Physalis* [1], [10], [11], [12]. However, phenotypic characters are generally influenced by environments and plant developmental stages [13]–[15]. In addition, species with similar morphological characters can not be easily distinguished [1]. Molecular markers are independent of environmental conditions and show higher levels of polymorphisms. They have been widely used in phylogenetic analysis in many organisms [16]–[20]. DNA sequences from few genes and ISSR markers have also been used to investigate the phylogeny of *Physalis* and their relationship to other genera in the Solanaceae family [1], [12], [21], [22]. Very recently, 5971 SSR markers were discovered through analyzing the assembled *P. peruviana* leaf transcriptome sequences [23], [24]. However, only 30 markers have publicly available primer information. The lack of available markers prevents the detailed study of the genetic diversity and phylogeny in *Physalis* at molecular level.

Tomato (*Solanum lycopersicum*) is a relative to *Physalis* in the same family. More than 3000 molecular markers have been developed since 1986 when the restriction fragment length polymorphism (RFLP) markers were used to construct the first tomato linkage map [25], [26]. The tomato genome has also been sequenced and publicly available [27]. Approximately 4000 genes conserved between tomato and *Arabidopsis* have been discovered [28]–[30]. These conserved ortholog sets (COS) have been used to analyze genetic diversity and phylogenetics in Solanaceae [31]–[33] and other families [34]–[36]. All these provide a potential to understand the genetics of *Physalis* at molecular level. Therefore, the objectives of the present study were to determine whether tomato markers can be used to amplify DNA fragments for investigating genetic diversity in *Physalis*, and to infer the relationships between tomato and *Physalis* using molecular marker and sequence data.

## Materials and Methods

### Plant materials

A collection of 38 accessions of *Physalis* (Table 1) originated from 11 countries were subjected to genetic diversity analysis and estimation of genetic relationship to tomato using bioinformatics and molecular markers. The tomato variety OH88119 and *S. lycopersicum* var. *cerasiforme* accession PI435238 were used as out-group controls for DNA amplification using tomato markers and phylogenetic analysis. Seeds of 36 *Physalis* accessions and PI435238 were kindly provided by Northeast Regional PI Station at Geneva, New York, USA. Seeds of Heirloom Purple and Toma Verde Green were purchased from a supermarket in the United States. Based on the information obtained from the website of Northeast Regional PI Station, 30 of 38 *Physalis* accessions belong to 6 species including *P. acutifolia, P. angulata, P. nicandroides, P. peruviana, P. philadelphica, P. pubescens*, while the species information for the remaining are not clear (Table 1). All seeds were sown in 128 Square Plug Tray Deep filled with a mixture of peat and vermiculite (3:1) in the greenhouse. Plants were grown in a protected greenhouse for DNA isolation.

**Table 1:** Information for 38 accessions of the genus *Physalis* and two tomato lines used in this study.

Accession	Species	ID	Origin	Comments
PI 270459	*Physalis philadelphica*	G 3493	Mexico	
PI 290968	*Physalis philadelphica*	G 13134	Argentina	not *Physalis philadelphica*
PI 291560	*Physalis philadelphica*	Tomatillo	India	
PI 512005	*Physalis philadelphica*	Tomatillo	Mexico	
PI 309812	*Physalis philadelphica*	Tomate	Mexico	
PI 360740	*Physalis philadelphica*	G22124	Ecuador	not *Physalis philadelphica*
PI 512009	*Physalis philadelphica*	Popo or Poposokol	Mexico	
PI 512008	*Physalis philadelphica*	Poposkuli (sg.), Voposkuli (pl.)	Mexico	
PI 512007	*Physalis philadelphica*	Tomatillo	Mexico	not *Physalis philadelphica*
PI 512006	*Physalis philadelphica*	Tomatillo	Mexico	
PI 512011	*Physalis philadelphica*	Tomatillo	Mexico	
PI 512010	*Physalis philadelphica*	Tomate	Mexico	
G 30318	*Physalis philadelphica*	Rendidora	United States	
G 32538	*Physalis philadelphica*	03V0204	Mexico	
G 32535	*Physalis philadelphica*	03V0201	Mexico	
G 32531	*Physalis philadelphica*	03V0197	Mexico	
G 32529	*Physalis philadelphica*	03V0195	Mexico	
G 32518	*Physalis philadelphica*	03V0184	Mexico	
G 32513	*Physalis philadelphica*	03V0179	Mexico	
G 32475	*Physalis philadelphica*	03V0140	Mexico	
G 32463	*Physalis philadelphica*	03V0128	Mexico	
G 32541	*Physalis philadelphica*	03V0207	Mexico	
PI 195810	*Physalis sp.*	Miltomate	Guatemala	*most likely Physalis philadelphica*
PI 343934	*Physalis sp.*	G 19531	Ethiopia	
PI 203942	*Physalis sp.*	No. 12313	Mexico	
PI 197692	*Physalis sp.*	Tomate Milpero	Mexico	*most likely Physalis philadelphica*
PI 197691	*Physalis sp.*	Tomate de Hoja	Mexico	*most likely Physalis philadelphica*
PI 194590	*Physalis sp.*	G 6457	Guatemala	*most likely Physalis philadelphica*
PI 291561	*Physalis peruviana*	-	India	
PI 285705	*Physalis peruviana*	-	Poland	
PI 232077	*Physalis peruviana*	Cape Gooseberry	South Africa	
PI 279231	*Physalis nicandroides*	No. 19521	Mexico	
PI 305457	*Physalis angulata*	G 14648	Sierra Leone	
PI 468103	*Physalis acutifolia*	G 26833	United States	
PI 644008	*Physalis pubescens*	Husk Tomato/Ground Cherry	United States	
PI 644010	*Physalis pubescens*	Plant Virus	Canada	
Heirloom purple	unknown	Heirloom Purple	unknown	
Toma Verde Green	unknown	Toma Verde Green	unknown	
PI 435238	*Solanum lycopersicum* var *cerasiforme*	No Known Plant ID	Colombia	
OH88119	*Solanum lycopersicum*	-	United States	

The information for all except the tomato line OH88119 were obtained from the website of Northeast Regional PI Station at Geneva, New York, USA (http://www.ars-grin.gov).

### Computational analysis of genes conserved between *Physalis* and tomato

Nucleotide sequences derived from the genus *Physalis* were obtained from the National Center for Biotechnology Information (NCBI, http://www.ncbi.nlm.nih.gov/) using the search and retrieval system for nucleotide data and phrase searching of *Physalis*. The results were then filtered with taxonomic group ‘Physalis’ (i.e. (physalis) AND "Physalis" [porgn:__txid24663]). Repetitions of sequences from the same gene were excluded using self-BLASTn approach with an expect value of less than e^−15^ and greater than 80% coverage of the query sequence. The unique sequences were then searched against the tomato ITAG Release2.3 Predicted CDS (SL2.40) downloaded from Sol Genomics Network (SGN, http://solgenomics.net/) [37] using BLASTn with an expect value of less than e^−10^ and greater than 80% coverage of the query sequence. The best hit of the tomato sequence for each *Physalis* sequence was then searched back to the *Physalis* sequence database to identify the reciprocal best match.

### Molecular Markers

Two sets of tomato DNA markers were used to estimate the relationship between *Physalis* and tomato. The first set of 60 COS markers were used for the initial test of the feasibility of using tomato markers in *Physalis*. These COS unigenes are conserved among several species including *Arabidopsis*, tomato, coffee, potato and pepper [28]–[30], and specifically developed for detecting polymorphisms in tomato [38]. After the initial test, three types of tomato markers (Table S1) including simple sequence repeat (SSR), potential intron polymorphism (PIP) and insertion/deletion (InDel) markers were randomly selected to test their ability to amplify DNA fragments in *Physalis*. The tomato SSR markers were developed by mining tomato expressed sequence tags (EST) database [39]. PIP was developed by comparing the conserved genes between *Arabidopsis* and tomato [30]. InDel markers could be divided into two groups. The first group was designated as ‘COS InDel’ because they were developed by comparing intronic region sequences of COS unigenes within cultivated tomatoes [38], [40], [41]. The second group was designated as ‘other InDel’ because they were developed by comparing DNA sequences of genes between two *Solanum* species [42], [43]. Most of these SSR, PIP, and InDel markers have been used to detect polymorphisms in cultivated tomatoes [38], [42]–[46]. More recently, Simbaqueba et al. [23] discovered a set of SSR markers through mining *P. peruviana* leaf transcriptome shotgun assembly (TSA) database and provided primer sequences of 30 SSR markers. These *P. peruviana* SSR markers were also used in this study.

### DNA isolation and marker analysis

Genomic DNA was isolated from young leaves collected from eight plants of each accession using the modified CTAB isolation method [47]. DNA quantity and quality were examined by running 5 μl of genomic DNA solution mixed with 3 μl loading buffer on a 1% agarose gel. The DNA was then diluted to a concentration of 5 ng.μl^−1^ for PCR amplification.

To test the feasibility of using tomato markers in *Physalis*, genomic DNA fragments from six *Physalis* accessions, Heirloom purple, PI232077, G32541, PI644010, PI203942, and PI512011, as well as the tomato lines PI435238 and OH88119 were amplified using 60 COS markers [38]. Genetic diversity in all *Physalis* accessions and two tomato lines were analyzed with 122 markers including 97 tomato markers (32 COS InDel, 26 SSR, 15 PIP, and 24 other InDel) and 25 SSR markers derived from *P. peruviana* (Table S1).

PCRs were conducted in a 10-μl reaction volume. Each reaction consisted of 10 mM Tris-HCl (pH 9.0 at room temperature), 50 mM KCl, 1.5 mM MgCl_2_, 100 μM each of dNTPs, 0.1 μM each primer, 10 ng of genomic DNA template, and 1 unit of *Taq* DNA polymerase. Reactions were heated at 94°C for 3 min followed by 36 cycles of 1 min at 94°C, 1 min at specific annealing temperature for each primer pairs (Table S1), and a 1-min extension at 72°C. Final reactions were extended at 72°C for 5 min. Amplification was performed in a programmable thermal controller (PTC-100; MJ Research, Inc., Watertown, MA). Following the amplification reactions, the PCR products were separated on 7% polyacrylamide gel and visualized using silver-staining approach as described in Chen et al. [44].

### Data collection and analysis

The presence or absence of each single fragment was coded by 1 or 0, respectively, and scored for a binary data matrix. Allele frequency of each marker was calculated for each accession. Nei’s genetic distance [48] were calculated for each pair of accessions using the program in the software package NTSYSpc 2.11a [49]. Since approximately 60% of accessions belong to *P. philadelphica*, the genetic distance and allele information for this species were also calculated. Unweighted Pair Group Method with Arithmetic Mean (UPGMA) cluster analysis was performed to develop a dendrogram.

Although the 38 accessions were from at least six species of *Physalis*, model without prior population information was used to assign individuals to population using a free software package of STRUCTURE2.2 [50]–[52]. Number of populations (*K*) was determined following the instruction in Pritchard et al. [50] with a burn-in period of 100,000 iterations and Markov Chain Monte Carlo of 100,000. Twenty independent runs were done for *K* varying from 1 to 10. The *K* optimum was defined according to the method proposed by Evanno et al. [53].

## Results

### Genes conserved between *Physalis* and *Solanum lycopersicum*

The total number of nucleotide sequences for *Physalis* obtained from NCBI were 34616, of which 33916 (98.0%) were from the leaf TSA of *P. peruviana*, while the remaining sequences were from at least 30 species including *P. philadelphica* (78 sequences). Self-BLASTn indicated that 11436 sequences were redundant. Of the remaining 23180 sequences, 19044 could be aligned to the tomato CDS with an expect value of less than e^−10^. However, only 4509 sequences matched to the tomato CDS by adding the parameter of greater than 80% coverage of the query sequence. Among the 4509 sequences, 372 matched to multiple (2–8) tomato CDS, while 811 matched to the same tomato CDS as others. Reciprocal blasting the *Physalis* sequence database using the best hits of tomato sequences resulted in 3356 genes with single copy in the TSA. These genes were considered as single-copy orthologous genes between *Physalis* and tomato. According to the GO terms assigned to the *Physalis* genes [24], 2200 genes belonged to Molecular Function class, 414 genes belonged to Biological Process class, 248 genes belonged to Cellular Component class, and 494 genes had no GO terms yet. In addition, 4867 sequences in *Physalis* did not match to any tomato CDS.

### Success of PCR amplification of DNA fragments in *Physalis*

The COS markers developed by comparing *Arabidopsis* and tomato sequences had a high rate of PCR success in amplification of DNA fragments in *Physalis*. Of the 60 COS markers (marker information not shown) used for initial test and the 15 PIP markers for genetic diversity analysis, 59 and 15 could amplify DNA fragments from at least one accession of *Physalis*, respectively. However, the COS markers developed by comparing sequences between tomato and other crops (COS InDel) or specifically for tomato (other InDel and SSR markers) had a low rate of PCR success between 65.4–75.0% (Table 2). Meanwhile, of the 30 SSR markers developed from *P. peruviana*, five could not amplify any DNA fragments from all accessions. The remaining 25 could amplify DNA fragments from at least one accession of *Physalis* and 40% had amplicons from tomato lines (Table 2). The average numbers of alleles in *Physalis* amplified by tomato and *P. peruviana* markers were 3.2 and 4.4, respectively. In total, 96 markers could amplify DNA fragments from at least one accession in *Physalis*. These results suggested that markers could be transferred between tomato and *Physalis*.

**Table 2:** PCR successes and number of alleles amplified in 38 accessions of the genus *Physalis* and two tomato lines.

Marker origin	Marker type	No. of markers	No. of markers with PCR success		No. of markers specific to		Total No. of alleles amplified		No. of alleles specific to		No. of shared alleles
			tomato	*Physalis*	tomato	*Physalis*	tomato	*Physalis*	tomato	*Physalis*	
*Solanum lycopersicum*	COS InDel	32	32	21	11	-	38	62	23	47	15
	other InDel	24	24	18	6	-	32	43	15	26	17
	PIP	15	15	15	0	-	18	53	4	39	14
	SSR	26	26	17	9	-	38	67	24	53	14
Sub-total		97	97	71	26	-	126	225	66	165	60
											
*Physalis peruviana*	SSR	25	10	25	-	15	12	111	1	100	11
											
Total		122	107	96	26	15	138	336	67	265	71

### Marker polymorphisms in *Physalis*

A high ratio of polymorphisms at marker level was observed in *Physalis*. Of the 96 markers having amplicons, 89 (92.7%) showed polymorphisms in *Physalis* and 71 (74.0%) showed polymorphisms in *P. philadelphica* (Table 3). All PIP markers could detect polymorphisms in *Physalis* and 66.7% of PIP markers detected polymorphisms in *P. philadelphica*. The COS InDel markers showed a relatively low ratio of polymorphisms in both *Physalis* (50%) and *P. philadelphica* (34.4%). All the 25 SSR markers derived from *P. peruviana* could detect polymorphisms in *Physalis*, while 21 showed polymorphisms in *P. philadelphica* (Table 3).

**Table 3:** Number of polymorphic markers and alleles amplified from 38 *Physalis* accessions and 25 *P. philadelphica* accessions.

Marker origin	Marker type	Total No. of markers	No. of polymorphic markers		No. of polymorphic alleles	
			in *Physalis*	in *P. philadelphica*	in *Physalis*	in *P. philadelphica*
*Solanum lycopersicum*	COS InDel	32	16	11	57	34
	other InDel	25	17	16	39	26
	PIP	15	15	10	50	29
	SSR	25	16	13	62	38
Sub-total		97	64	50	208	127
						
*Physalis peruviana*	SSR	25	25	21	111	69
Total		122	89	71	319	196

The accessions with ambiguous species information were excluded from *P. philadelphica* cluster for calculation.

A total of 336 alleles with an average of 3.5 alleles per marker were amplified by the 96 markers in *Physalis*. The number of alleles amplified by each marker varied from 1 to 5 (Table S1). However, the average number of alleles for each marker was 1.4 for each accession (data not shown). The total polymorphic alleles were 319 and 196 with averages of 3.6 and 2.8 in *Physalis* and *P. philadelphica*, respectively (Table 3). The average numbers of alleles in *Physalis* (4.4) and *P. philadelphica* (3.3) generated by *P. peruviana* SSR markers were higher than those generated by tomato markers (3.3 in *Physalis* and 2.5 in *P. philadelphica*).

### Genetic diversity in *Physalis*

**Figure 1:**
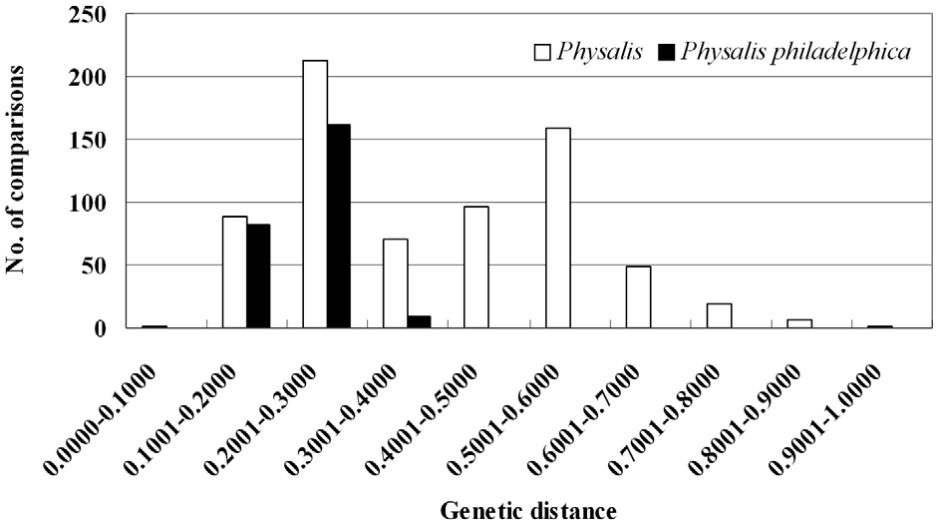
Distribution of Nei's genetic distance values. The genetic distance was obtained from pair wise comparisons of 38 accessions of *Physalis* and 23 accessions of *P*. *philadelphica* with molecular marker data using the software NTSYSpc 2.11a.

The average Nei's genetic distance was 0.3806 with a range from 0.2865 (G32475) to 0.7091 (PI343934) for each accession in *Physalis*. The largest genetic distance (0.9006) was between accessions PI343934 and PI644010, while accessions PI232077 and PI291561 had the least genetic distance of 0.0768. The distribution of genetic distance between any two accessions had two peaks at 0.2001–0.3000 and 0.5001–0.6000, respectively (Fig. 1). The genetic distance between species pairs in *Physalis* ranged from 0.3967 to 0.8534 (Table 4). The lowest was between *P. angulata* and *P. acutifolia, while the highest was for* PI343934 (*P. sp.*) and *P. pubescens*. The *Physalis sp*. accession PI343934 had the largest genetic distance to all six species with a range of 0.7080 to 0.8534, while the *Physalis sp*. accession PI203942 had large genetic distance to four species but a relatively small genetic distance to PI343934 (0.4876) and *P. philadelphica* (0.4969). Since approximately 60% of the accessions belong to *P. philadelphica*, the genetic distance within this species was also calculated. The genetic distance between any two accessions in *P. philadelphica* varied from 0.1102 (G32475 vs G32538) to 0.3437 (PI512006 vs G30318), while the average genetic distance for each accession ranged from 0.1854 (PI512010) to 0.2814 (G32513) with an overall average of 0.2241.

**Table 4:** The Nei's genetic distance between species pairs in *Physalis*.

	*P. acutifolia*	*P. angulata*	*P. nicandroides*	*P. peruviana*	*P. philadelphica*	*P. pubescens*	PI343934 (*P. sp.*)
*P. angulata*	0.3967						
*P. nicandroides*	0.5679	0.5214					
*P. peruviana*	0.5071	0.6004	0.4920				
*P. philadelphica*	0.5826	0.5919	0.5237	0.4409			
*P. pubescens*	0.5280	0.4426	0.4069	0.4552	0.5394		
PI343934 (*P. sp.*)	0.8054	0.7893	0.7818	0.8273	0.7080	0.8534	
PI203942 (*P. sp.*)	0.6090	0.6099	0.5774	0.6012	0.4969	0.6208	0.4876

**Figure 2:**
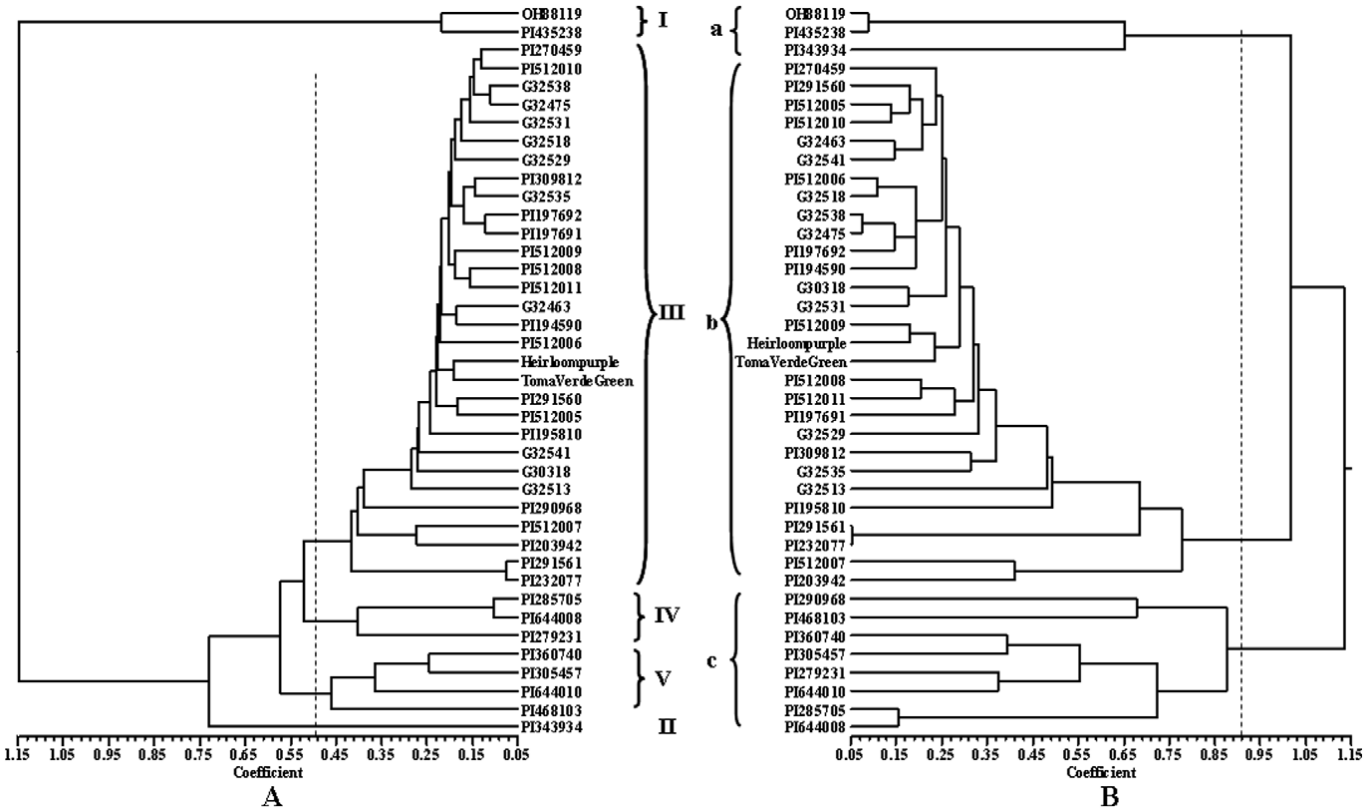
Dendrogram of 38 *Physalis* accessions based on all marker data (A) and *P. peruviana* SSR marker data (B). The dendrogram was generated from Nei’s genetic distance matrix by UPGMA in NTSYSpc 2.11a. Tomato lines OH88119 and PI435238 were used as out-group controls.

To test whether there was a difference between dendrograms generated by markers from tomato and *P. peruviana*, three dendrograms were separately created using only tomato marker data, only *P. peruviana* marker data and both marker data. UPGMA cluster analysis showed that dendrograms obtained using only tomato marker data (data not shown) and all marker data (Fig. 2A) were very similar. Two tomato lines OH88119 and PI435238 formed a cluster (I) that was distinct from *Physalis* accessions. *Physalis sp*. accession PI343934 was also far away from other accessions and thus could be considered as a separate cluster (II). At the genetic distance of 0.5000, the remaining 37 *Physalis* accessions could be grouped into three clusters (III, IV, and V). Cluster III was the largest one included almost all *P. philadelphica* accessions, five *Physalis sp*. accessions, and two *P. peruviana* accessions. Cluster IV included three accessions PI285705 (*P. peruviana*), PI644008 (*P. pubescens*), and PI279231 (*P. nicandroides*). Cluster V contained four accessions PI360740 (*P. philadelphica*), PI305457 (*P. angulata*), PI644010 (*P. pubescens*), and PI468103 (*P. acutifolia*). The dendrogram created using the 25 *P. peruviana* SSR marker data showed the same pattern (Fig. 2B). However, only three main clusters were observed. The *Physalis sp*. accession PI343934 was grouped into the same cluster (a) as tomato lines. The accessions in cluster b were almost the same as those in cluster III described above except the accession PI290968 was grouped into the cluster c. Cluster c included seven accessions in the clusters IV and V described above as well as PI290968.

### Population structure in *Physalis*

**Figure 3:**
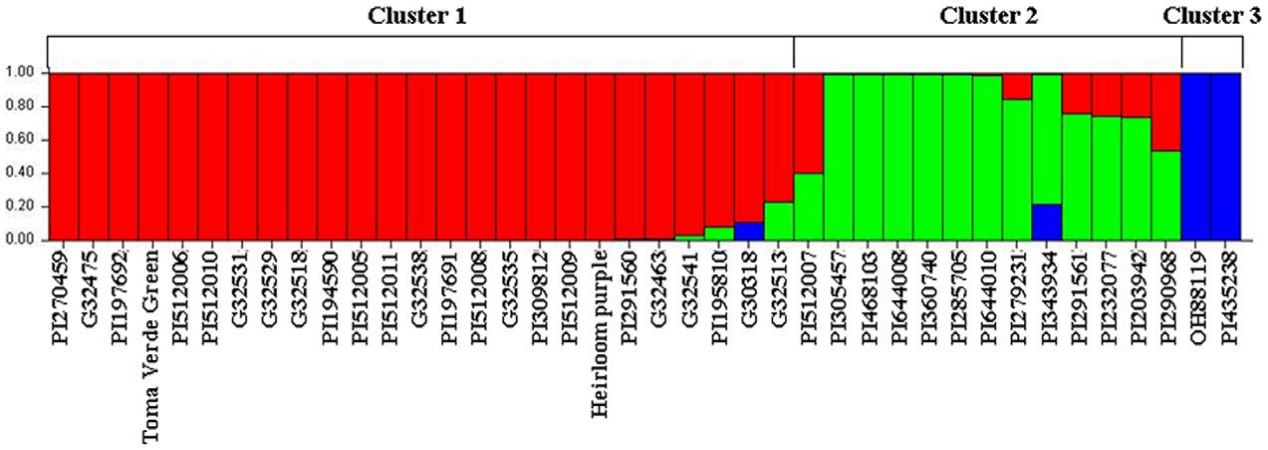
Population structure of 38 *Physalis* accessions and two tomato lines using STRUCTURE software and 122 markers. The coefficients of estimated ancestry per accession in each cluster were represented by an individual bar, where each color refers to a distinct cluster. The name of the accession is below the bar.

Without specifying prior information concerning species and allowing for admixed individuals, we tested population structure for *K* = 1 to 10. From the summary plot of membership coefficients (*Q*), it was clear that the model with *K* = 1 was completely insufficient to model the data. It was not unexpectedly that the two tomato lines were always grouped into one cluster and were separated from *Physalis* When *K*>1. Of course, two tomato lines formed one cluster, while the *Physalis* accession formed another cluster when *K* = 2. When *K* increased from 3 to 10, 25 *Physalis* accessions always clustered together (cluster 1, Fig. 3), while the remaining 13 *Physalis* accessions could form one cluster when *K* = 3 (cluster 2, Fig. 3) and up to five clusters when K = 10. However, the best number of clusters was 3 (Fig. S1), which was supported by the plateau observed for parameter P(*X*|*K*) at *K* = 3 (Fig. S2), which was consistent with the UPGMA clusters (Fig. 2). The 25 accessions in cluster 1 were from the cluster III (Fig. 2A) or cluster b (Fig. 2B). Cluster 2 included all eight accessions in clusters II, IV, and V (Fig. 2A). Five accessions, PI512007, PI291561, PI232077, PI203942, and PI290968 from cluster III (Fig. 2A) or cluster b (Fig. 2B) had less alleles from cluster 1, and thus were grouped into cluster 2 (Fig. 3). Indeed, they were close to cluster IV (Fig. 2A) or cluster c (Fig. 2B) in UPGMA analysis.

## Discussion

Here we compared the DNA sequences between *Physalis* and tomato, and presented the use of tomato markers in genetic analysis in the genus *Physalis*. A total of 3356 unigenes accounting for 14.5% sequences analyzed in *Physalis* were identified as single-copy orthologous genes between tomato and *Physalis*, which was lower than the numbers of COS genes identified between tomato and other genera including potato, pepper, coffee in the same family [29]. This could be due to the following two reasons. First, previous studies [28], [29] used only one parameter, the E-value, to identify orthologous genes. Here we used two parameters, a moderate E-value of e^−10^ and 80% sequence coverage. This strategy excluded a number of genes having low E-value but not enough sequence coverage. The number of total hits increased from 4509 to 7498 when the percentage of sequence coverage was decreased from 80% to 70%, which resulted in the increase of the number of orthologous genes subsequently. Second, the TSA data of *Physalis* only includes transcriptome sequences from leaf sample [24]. Lack of sequences for a number of genes specifically expressed in other organs or tissues prevents genome-wide identification of orthologous genes between tomato and *Physalis*, which could also result in the low number of orthologous genes identified in this study. This could be supported by molecular marker data. Of the 97 tomato markers evenly distributed on 12 tomato chromosomes used in this study, 54 could amplify DNA fragments from the three *P. peruviana* accessions, while 10 of 25 SSR markers derived from *P. peruviana* could amplify DNA fragment from the two tomato lines. These results indicated that molecular markers developed for tomato could be used for genetic study in *Physalis*.

High diversity at morphological level is observed in wildly distributed species or those that are managed and cultivated in the genus *Physalis*. However, due to the lack of molecular markers, little is known about the genetics of *Physalis* at molecular level. A recent study analyzed 12 populations of eight species from Mexico with six ISSR primers and discovered high polymorphisms among species at molecular level [12]. All bands amplified by the six ISSR primers were polymorphic among the eight species. On the contrary, only 22% SSR markers derived from *P. peruviana* showed polymorphisms between *P. peruviana* and *P. floridana* [23]. In this study, the SSR markers derived from *P. peruviana* revealed high level of polymorphisms among species. All 25 markers showed polymorphisms in *Physalis* and 84% markers showed polymorphisms with *P. philadelphica* (Table 3), which was inconsistent with the finding from Simbaqueba et al. [23]. The sample size and divergence might be the cause of this difference. Only seven accessions in *P. peruviana* and one accession in *P. floridana* were used in Simbaqueba et al. [23], while 38 accessions from at least six species were investigated here. The high level of polymorphisms was also supported by using the markers derived from tomato, with 92.7% and 74.0% markers that were polymorphic in *Physalis* and *P. philadelphica*, respectively. All these suggested that a broad genetic diversity at DNA level also existed in wild and cultivated species of *Physalis*, which could be due to their self-incompatibility [54].

However, the existence of high morphological variation makes taxonomic identification more challenging in *Physalis*. Similar morphology as well as lack of detailed field notes of collections and experiments result in the estimation of the number of species in the genus varying from 75 to 120 [1]. Although DNA sequences of few genes have been used in phylogenetic analysis [21], [22], the relationships of species in *Physalis* remain unclear. Based on morphological characters, 30 accessions used in the present study belong to 6 species, while the species information for the remaining eight accessions is unknown (Table 1). With the marker data obtained in this study, six of them (PI197691, PI197692, PI194590, PI195810, Heirloom purple, and Toma Verde Green) could be assigned to the species *P. philadelphica*. The accession PI203942 was also close to the *P. philadelphica* cluster. The accession PI343934 stood alone. Meanwhile two accessions previously identified as *P. philadelphica* could not be assigned to the *P. philadelphica* cluster. Two *P. peruviana* accessions, PI291561 and PI232077, were close to the *P. philadelphica* cluster but not in the same cluster as the other *P. peruviana* accession PI285705. Two *P. pubescens* accessions PI644008 and PI644010 were not in the same cluster either (Fig. 2). Structure analysis suggested that some of these accessions might have alleles outside its own species (Fig. 3). The results obtained here suggested that it was necessary to take molecular approach to characterize species in the genus *Physalis*.

Previous study using six ISSR markers to analyze genetic variation in eight species of *Physalis* found that genetic interspecific similarity values ranged from 0.20 to 0.57, and intraspecific similarity values ranged from 0.55 to 0.71. The genetic distance in *P. philadelphica* was 0.37 [12]. Same trend was also observed in the present study. The range of Nei’s genetic distance between individual accessions was larger in *Physalis* (0.0768–0.9006) than in *P. philadelphica* (0.1102–0.3437), *P. peruviana* (0.0768–0.4549) and *P. pubescens* (0.4177). These results indicated that the genetic variation within a species was less than that in the genus *Physalis*. Majority (71.4%) of the genetic distances between species were greater than 0.5000. The genetic distances between the *Physalis sp*. accession PI343934 and all six species were among the largest, while that between *P. angulata* and *P. acutifolia* was the smallest (Table 4). However, it might not reflect the true genetic relationships between these species because only one accession from each of three species *P. nicandroides*, *P. angulata* and *P. acutifolia*. More accessions are needed to understand the genetic relationships among species at molecular level.

It has been suggested that domestication and inbreeding dramatically reduced the genetic variation in tomato [46], [55]–[57]. In *Physalis*, although the fruits of more than 10 wild species grown in natural areas and traditional agroecosystems are collected for consumption, only the species *P. philadelphica* (tomatillo) has been domesticated and cultivated [12]. In this study, of the 319 polymorphic alleles amplified in *Physalis*, 196 (61.4%) showed polymorphisms in *P. philadelphica*. This result suggested that domestication reduced the genetic variation in cultivated species though the reduction was not as severe as in cultivated tomato, which might also be due to their self-incompatibility. Cultivated tomato is self-compatible though several wild species are self-incompatible [58]. Due to domestication, selection and no exchange of genetic information with the wild germplasm for a long time [56], the genomes of tomato cultivars contain less than 5% of the genetic variation of their wild relatives [59]. By contrast, most genotypes of tomatillo posses gametophytic self-incompatibility and thus are obligate outcrossers [54]. Although *P. philadelphica* has been domesticated for centuries, the wild forms are frequently found growing in cultivated fields in traditional agricultural systems [60]. Domestication has had little effect on overall levels of tomatillo diversity nonetheless fruits in domesticated varieties are up to 15 times larger than wild fruits [61]. However, wild forms does harbor diversity not found in cultivated types [61]. Therefore, it is essential to pay attention to the reduction of genetic variation in *Physalis*.

## Supporting Information

Figure S1 **Estimation of optimum number of clusters (** ***K*** **) using the method described in Evanno et al.** [**52**] **.** Δ*K* is calculated as the mean of the absolute values of *L’’*(*K*) averaged over 20 runs divided by the standard deviation of *L*(*K*). Δ*K*  =  *m*(|*L’’*(*K*)|)/*s*[*L*(*K*)], which expands to Δ*K*  =  *m*(|*L*(*K*+1)-2*L*(*K*)+*L*(*K*-1)|)/*s*[*L*(*K*)]. *L*(*K*) is the Pr(*X*|*K*) referred as ‘Ln P(D)’ in the output of STRUCTURE software.
(TIF)

Figure S2 **The graph for the parameter** ***L*** **(** ***K*** **) and number of clusters (** ***K*** **).** The plateau is achieved at *K* = 3. Although the log likelihood *L*(*K*) is still increasing, an increase of the variance of *L*(*K*) between runs is also observed when *K* is greater than 3. Thus, the optimum number of clusters for 38 *Physalis* accession and two tomato lines is 3.
(TIF)

Table S1 **Marker information used for PCR amplification and genetic diversity analysis in 38 accessions from the genus** ***Physalis*** **.**
(XLS)
