# Supplementary figures and images for: Identification of Single-Copy Orthologous Genes between Physalis and Solanum lycopersicum and Analysis of Genetic Diversity in Physalis Using Molecular Markers
Source: PLoS One. 2012 Nov 16;7(11):e50164. doi: 10.1371/journal.pone.0050164 (PMC3500348; doi:10.1371/journal.pone.0050164)

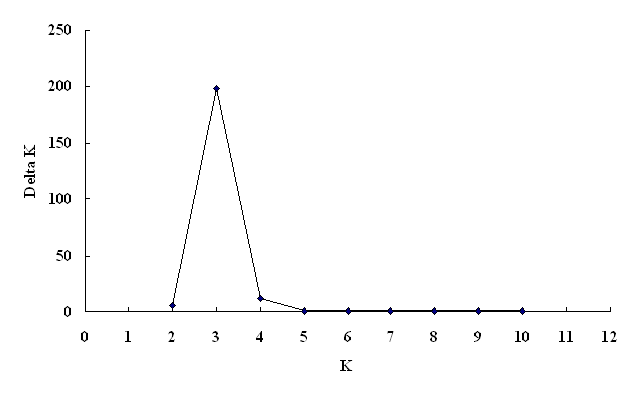

Supplement: Figure S1 — Estimation of optimum number of clusters ( K ) using the method described in Evanno et al. [52] . ΔK is calculated as the mean of the absolute values of L’’(K) averaged over 20 runs divided by the standard deviation of L(K). ΔK = m(|L’’(K)|)/s[L(K)], which expands to ΔK = m(|L(K+1)-2L(K)+L(K-1)|)/s[L(K)]. L(K) is the Pr(X|K) referred as ‘Ln P(D)’ in the output of STRUCTURE software. (TIF) [file pone.0050164.s001.tif]

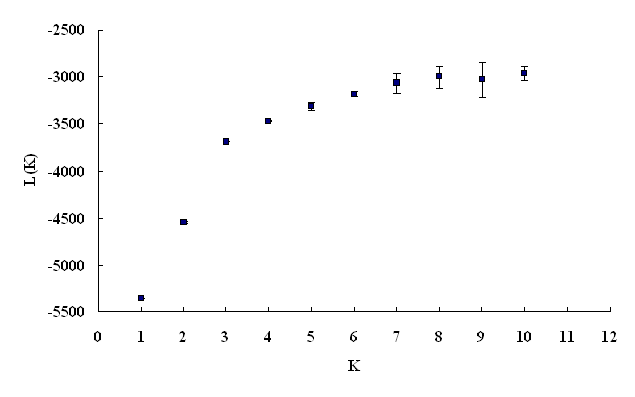

Supplement: Figure S2 — The graph for the parameter L ( K ) and number of clusters ( K ). The plateau is achieved at K = 3. Although the log likelihood L(K) is still increasing, an increase of the variance of L(K) between runs is also observed when K is greater than 3. Thus, the optimum number of clusters for 38 Physalis accession and two tomato lines is 3. (TIF) [file pone.0050164.s002.tif]
